# Mixed Fungal Infection (*Aspergillus*, *Mucor*, and *Candida*) of Severe Hand Injury

**DOI:** 10.1155/2014/954186

**Published:** 2014-03-13

**Authors:** Milana Obradovic-Tomasev, Aleksandra Popovic, Nada Vuckovic, Mladen Jovanovic

**Affiliations:** ^1^Clinic of Plastic and Reconstructive Surgery, Clinical Center of Vojvodina, Hajduk Veljkova 1, 21000 Novi Sad, Serbia; ^2^Department of Surgery, Faculty of Medicine of Novi Sad, University of Novi Sad, 21000 Novi Sad, Serbia; ^3^Institute of Pathology, Faculty of Medicine of Novi Sad, University of Novi Sad, 21000 Novi Sad, Serbia

## Abstract

Severe hand injuries are almost always heavily contaminated and hence wound infections in those patients are frequent. Fungal wound infections are rare in immunocompetent patients. A case of mixed fungal infection (*Aspergillus*, *Mucor*, and *Candida*) was documented in a young male patient, with a severe hand injury caused by a corn picker. The diagnosis of fungal infection was confirmed microbiologically and histopathologically. The treatment was conducted with repeated surgical necrectomy and administration of antifungal drugs according to the antimycogram. After ten weeks the patient was successfully cured. The aggressive nature of *Mucor* and *Aspergillus* skin infection was described. A high degree of suspicion and a multidisciplinary approach are necessary for an early diagnosis and the initiation of the adequate treatment. Early detection, surgical intervention, and appropriate antifungal therapy are essential in the treatment of this rare infection that could potentially lead to loss of limbs or even death.

## 1. Introduction

Molds such as* Aspergillus* and* Mucor* are the most common opportunistic filamentous fungi, which can cause very serious infections that develop rapidly and that can sometimes be fatal. Generally these infections occur in immunocompromised patients, patients with unregulated diabetes, and patients treated with immunosuppressive drugs. Rare cases of these types of fungal infections in previously healthy immunocompetent patients are documented. The infection is caused by spores from the environment, which get into the body through the lungs, gastrointestinal system, or the skin. Primary infection of the skin may occur only when the skin is damaged [1, 2]. Here we present the case of a young farmer with a hand injury sustained on a farm, where a mixed infection caused by bacteria and fungi developed and was successfully cured.

## 2. Case Report 

**Figure 1:**
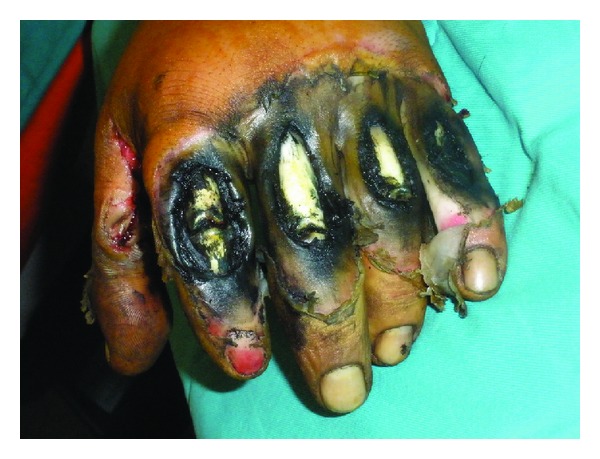
Left hand corn picker injury at admission to the hospital.

**Figure 2:**
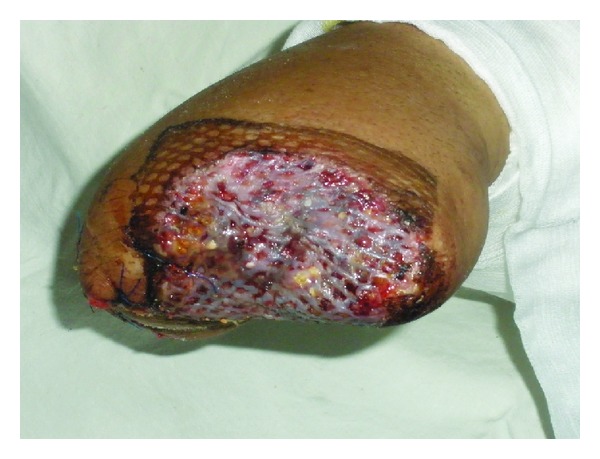
Eighth day after injury: initial signs of graft loss.

**Figure 3:**
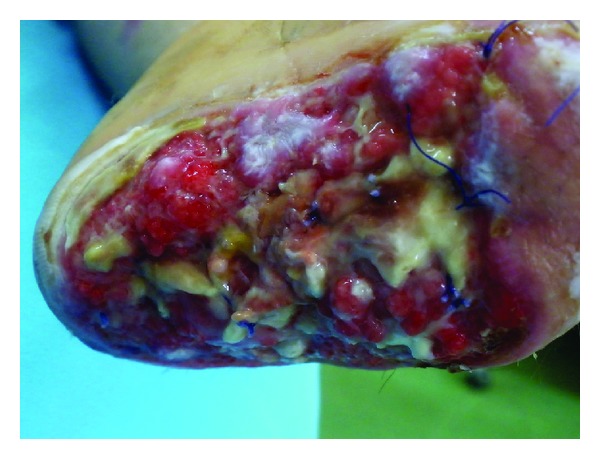
Ninth day after injury: complete graft loss and dark necrotic tissue.

**Figure 4:**
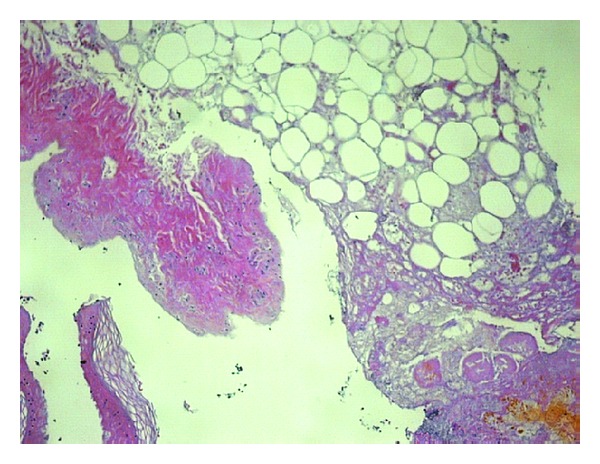
Fragment of necrotic skin with subcutaneous fat tissue and epidermis with subepidermal cleft (HE ×100).

**Figure 5:**
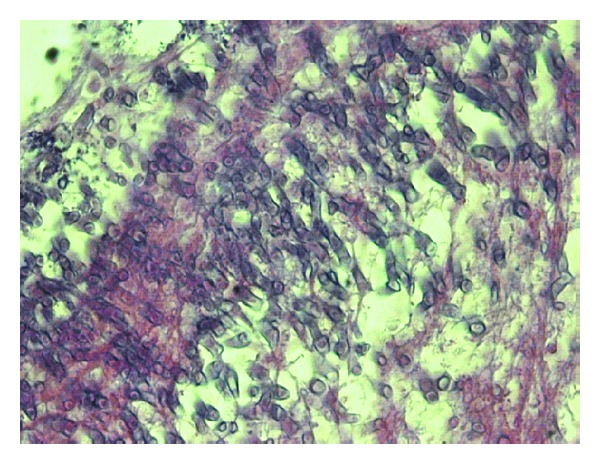
Fungal colonies on the surface of the necrotic mass with broad nonseptated hyphae that branch (HE ×630).

**Figure 6:**
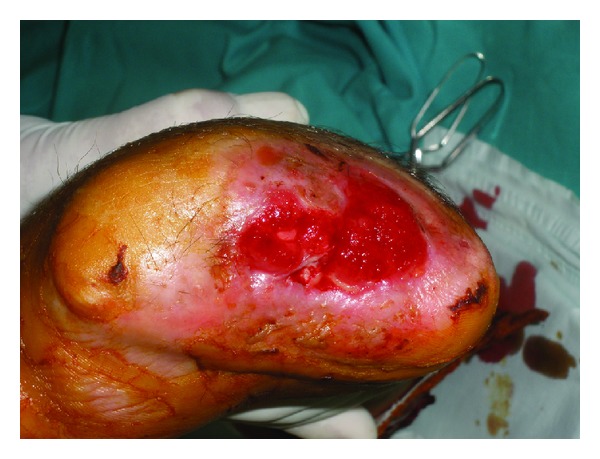
Forty days after injury: no signs of infection.

**Figure 7:**
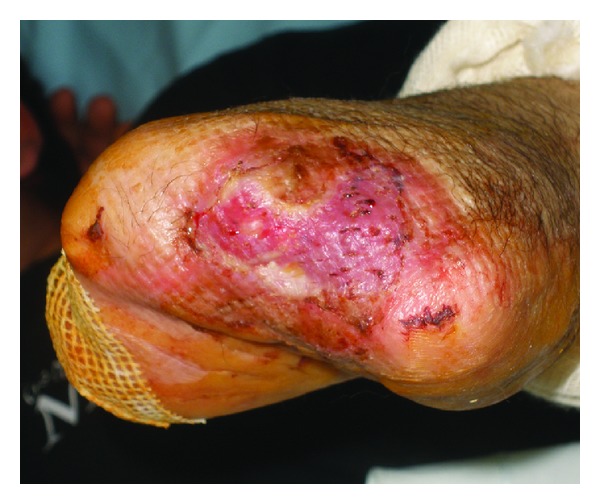
Full acception of the skin graft one week after transplantation.

A 28-year-old male patient was admitted to the hospital with a severe left hand injury caused by a corn picker (Figure 1). The patient was a young and healthy person. He underwent an operation within the first 6 hours after the injury occurred. Amputation of the I–V fingers was done in general anesthesia and defects of the skin were covered with split-thickness skin grafts. Wound toilet was conducted with povidone iodine. Upon admission, the patient received antitetanus and antibiotic prophylaxis (ceftriaxone 2 × 1 gr, amikacin 2 × 500 mg, and metronidazole 3 × 500 mg). The operative and early postoperative course were normal. In the further postoperative period the patient was in good general condition, with normal body temperature, with normal laboratory results and microbiological findings of the wound swabs. Local findings were also normal; wounds were clean and dry; the grafts were well fitted, pink, and with no secretion; after 7 days the grafts were fully attached to the whole surface. The dressing of the wounds was conducted with greasy gauze and povidone iodine compresses. On the eighth day of the regular dressing a scanty turbid secretion appeared in the wound, and circumscripted yellowish orange fields appeared on the skin grafts. A fungal infection was suspected (Figure 2). The wound swabs were taken for microbiological analysis. Antibiotics were suspended. Dressing of the wounds was done twice a day with topical application of miconazole cream.* Enterococcus *and* Candida *spp. in large numbers and* Aspergillus *sp. in small numbers were isolated from the wound swab. The next day, there was almost a complete loss of skin grafts with visible plaques of very dark brown granulation tissue necrosis (Figure 3). Again, swabs were obtained and a biopsy was taken for histopathological analysis. The dressing of the wounds was still conducted twice a day with miconazole. Swab analysis showed that next to the* Enterococcus* and* Candida *spp. there was* Aspergillus *sp. in large numbers. Histological examination showed fragments of connective tissue with preserved morphology, fat tissue saturated with granulocytes and zones of necrosis, necrotic stratified squamous epithelium, many granulocytes, colonies of bacteria (cocci), and colonies of fungi (hyphae and spores). According to the antimycogram and in consultation with the infectious disease specialist, it was determined that further therapy was to include amphotericin B (250 mg/kg/24 h). One week after the infection occurred voriconazole was included with an initial dose of 2 × 200 mg and then 200 mg a day for three weeks. Wound swabs were obtained twice a week; during the first week a biopsy was taken three times. Histological findings of the fifth day of the infection correspond to skin necrosis with the presence of fungi-zygomycetes; the differential diagnosis in the first place is* Mucor*. Microscopically, skin with large areas of necrosis, covered with dense necrotic detritus, with fibrin and inflammatory infiltrate (Figure 4). In the necrotic masses colonies of bacteria and fungi are clearly visible. Hyphae are broad and long, measuring from about 20 to 30 microns, without septa, branching at an angle of up to 90 degrees. The edges have a darker color than the bright center (Figure 5). A week after the infection began, in the wound swab,* Enterococcus* and* Candida *spp. were isolated; after 12 days there was only colonization of* Enterococcus* and coagulase negative* Staphylococcus* spp. and two days after that the swabs were negative. Blood cultures were negative. The blood analysis showed that sedimentation rate was moderately accelerated for three weeks after the infection occurred (first hour 22 mm/h) and slightly elevated gamma GT (up to 73 U/L). During the whole time the patient was in a good general condition and with normal body temperature. Wound treatment was carried out by repeated necrectomy and dressing with miconazole and compresses of calcium alginate with silver. After the swabs were negative and the skin defects were covered with healthy granulation tissue (Figure 6), transplantation of free skin grafts was carried out. The grafts were fully accepted one week after (Figure 7). There was no proximal propagation of the infection and no need for reamputation. The patient was discharged from the hospital 51 days after the injury.

## 3. Discussion

Fungal infection is easily understood in immunocompromised patients with deficient phagocytosis. In cases of posttraumatic fungal infections (filamentous fungal infection) the existence of acidosis due to the large tissue damage and loss of vitality, with local immunodepression, may be the explanation for the development of this type of infection in previously healthy individuals [1, 2]. Risk factors for fungal infections are existence of immune deficiency, uncontrolled diabetes mellitus, myeloproliferative diseases, long-term use of corticosteroids, transplant patients, AIDS, intravenous drug users, and patients undergoing chemotherapy [3–5]. A large number of spores of these fungi are present in the soil. Most of these infections have been described in traffic and agricultural traumatism and in natural disasters [1]. For the fungal infection to develop there has to be an open wound in the skin through which the spores are inoculated into the tissue [3]. That is the reason why initial surgical treatment of wounds is very important, especially in the case of agricultural injuries. Wound toilet, the removal of any foreign material from the wound and its surroundings, disinfection, and debridement of dead tissue are essential. It should be noted that povidone iodine cannot eradicate inert spores from the patient, which may be the explanation for the subsequent development of the fungal infection [3]. These infections are characterized by the invasion of hyphae in healthy tissue and blood vessels and consecutive thrombosis and necrosis of the affected tissue, which is manifested by a local inflammatory reaction and the presence of mold colonies [1]. As a consequence of the infection progression sepsis may develop. In open skin injuries symptoms are usually manifested within 10 days after the injury [1]. Diagnosis is based on the histological examination of repeated tissue biopsies and the culture of wound swabs. The culture of wound exudates is not always reliable, because the results are positive in only 30% of histologically proven* Mucor* mycosis [4]. Clinically,* Mucor* mycosis is characterized by necrosis with a dark central part or by necrotizing cellulitis, while in the case of* Aspergillus* infection papules are present, nodules, and/or necrosis. Histologically,* Mucor* mycosis is presented with necrosis and invasion of blood vessels; hyphae are broad, not septated (rarely septated), and with a branching angle of 90°. Granulomatous inflammation and focal necrosis are present subcutaneously.* Aspergillus* infection histological finding corresponds with granuloma or abscess; hyaline dichotomous septated hyphae which are branching at an angle of 45° are seen; also, vascular invasion and occlusion are present [6]. Given the aggressive nature of this fungal infection it is essential to start with the appropriate treatment as soon as possible. Since the symptoms, clinical signs, and local findings are nonspecific, it is crucial to set a high level of suspicion for a fungal infection, immediately discontinue antibiotics, begin aggressive surgical debridement, set the right diagnosis, and initiate parenteral systemic antifungal therapy [4, 7, 8]. Early identification of fungi is important to initiate parenteral antifungal therapy. Antifungal therapy involves intravenous application of amphotericin B in the case of* Aspergillus* infection and in the case of* Mucor *mycosis [9]. Since 2002, in addition to amphotericin B, or in combination with it, other antifungal drugs as voriconazole, posaconazole, and so forth are also used [1]. Adequate surgical treatment is of great importance in posttraumatic infections. In these cases, there is a rapid spread of infection followed by expanding areas of necrosis where the penetration of antifungal drugs is difficult, all of which result in the alteration of the local tissue defense capability due to the tissue acidosis and abnormal phagocytic function. Aggressive debridement is one of the preconditions for a successful treatment.

The possibility of fungal infections should always be considered, especially in cases where the infection occurs a week or more after the injury, when there is a loss of skin grafts that were accepted, in the case of a patient with risk factors and patients with severe agricultural injuries. A high degree of suspicion and a multidisciplinary approach are necessary for an early diagnosis and the initiation of the adequate treatment. Early detection, surgical intervention, and appropriate antifungal therapy are essential in the treatment of this rare infection that could potentially lead to loss of limbs or even death.
